# Chitotriosidase and Neopterin as Two Novel Potential Biomarkers for Advanced Stage and Survival Prediction in Gastric Cancer—A Pilot Study

**DOI:** 10.3390/diagnostics13071362

**Published:** 2023-04-06

**Authors:** Vlad-Ionuţ Nechita, Nadim Al Hajjar, Cristina Drugan, Cristina-Sorina Cătană, Emil Moiş, Mihaela-Ancuţa Nechita, Florin Graur

**Affiliations:** 1 Department of Medical Informatics and Biostatistics, “Iuliu Hațieganu” University of Medicine and Pharmacy, Louis Pasteur Str., No. 6, 400349 Cluj-Napoca, Romania; nechita.vlad@umfcluj.ro; 2 “Octavian Fodor” Regional Institute of Gastroenterology and Hepatology, 010336 Cluj-Napoca, Romania; 3 Department of Surgery, “Iuliu Hațieganu” University of Medicine and Pharmacy, Croitorilor Str., No. 19–21, 400162 Cluj-Napoca, Romania; 4 Department of Medical Biochemistry, “Iuliu Hațieganu” University of Medicine and Pharmacy Cluj-Napoca, Louis Pasteur Str., No. 6, 400349 Cluj-Napoca, Romania; 5 “Ion Chiricuță” Oncology Institute, Republicii Str., No. 34–36, 400015 Cluj-Napoca, Romania

**Keywords:** gastric cancer, neopterin, chitotriosidase, stadialisation, resectability, survival

## Abstract

Gastric cancer is the fifth type of neoplasia most frequently diagnosed and the fourth cause of death among other cancers. Prevalence is around two times higher for males than females. Chitotriosidase and neopterin are two molecular biomarkers with potential diagnostic and prognostic use in malignant pathology. We conducted a longitudinal prospective cohort study on thirty-nine patients with gastric adenocarcinoma, with a male-to-female ratio of 1.78 and an average age of 64.3 ± 9.97 years. No statistically significant differences in biomarker levels at presentation were observed between curative-intent surgery (28 patients) and advanced cases, suited only for palliative procedures (11 patients). Biomarker values were not significantly different for the advanced T stage and the presence of metastasis (*p* > 0.05—Mann Whitney test). The patients that died in the first 30 days after surgery did not present significantly different values at baseline, in comparison with those that had longer survival times, though a significant cut-off value was observed for chitotriosidase activity at 310 nmol/mL/h [AUC (area under the curve) = 0.78; 95% CI (0.61–0.92)]. The cut-off values corresponding to death after the first year, tumor invasion, and metastasis were not statistically significant. In the COX multivariate model, neopterin did not validate itself as a prognostic biomarker, however, chitotriosidase activity before surgery was significantly associated with overall survival (HR = 1.0038, *p* = 0.03). We conclude that chitotriosidase may have the potential to improve the prognostic model for gastric adenocarcinoma.

## 1. Introduction

Gastric cancer is the fifth most frequently diagnosed type of neoplasia (5.6%) after mammary (11.7%), lung (11.4%), colorectal (10%), and prostate (7.3%) cancer, being the fourth cause of death due to neoplasia (after lung, colorectal, and liver cancer) [1]. Gastric cancer affects the male population about two times more frequently than females [1,2]. Higher incidence and prevalence of gastric cancer were observed in Eastern Asia and Eastern Europe, while in Northern America, Africa, and Northern Europe, the rates are lower [3].

Survival in gastric cancer is poor for advanced stages. Katai et al., working on a cohort of over 100,000 patients, reported a five years survival rate of 71.1%, 95% CI (70.9–71.3%) for patients with surgical resection. From 118,367 patients with gastrectomy, 587 died in the first 30 days after the intervention, leading to short-term postoperative mortality of 0.5%. For stages I and II, the five-year overall survival was above 68.9%; for stages IIIA and IIIB it was above 32.3%, while for stage IV it dropped to only 17%. The presence of metastasis reduces the five-year survival rate to 11.5% for liver metastasis and 9.5% for peritoneal metastasis [4]. According to Isobe et al., the 30 days postoperative mortality was 0.6% for patients who had undergone gastrectomy [5].

Chitotriosidase and neopterin are molecular biomarkers for cellular immune response activation. Activated macrophages are responsible for the secretion of both molecules [6,7]. Neopterin, a pteridine derivative that results after GTP (guanosine triphosphate) catabolism, is a product of monocyte and macrophage activation, after stimulation with gamma interferon, a proinflammatory cytokine [7,8,9].

Murr et al. suggested that malignant cells present a modified cell surface that can trigger specific cellular immune system activation and neopterin production. Otherwise, they considered this biomarker to be inadequate for screening or diagnostic purposes in malignant pathology, as the frequency of higher serum levels of neopterin is related to tumor type (with over 90% frequency of increased neopterin for hematologic neoplasms, such as Hodgkin and non-Hodgkin lymphoma, and less than 20% for breast cancer, respectively). On the other hand, the utility of neopterin can be relevant for the prognostic estimation at the moment of diagnosis, as the tumor stage can influence neopterin elevation [10].

According to Unal et al., neopterin levels for subjects with gastric cancer (15.26 ± 11.46 nmol/L) were significantly higher than for healthy age and gender-matched subjects in the control group (9.87 ± 2.90 nmol/L), without malignancy, infections, or inflammatory pathology [11].

Hacisevki et al. also suggested that neopterin levels (mean ± standard error) before intervention can be a possible biomarker for gastrointestinal tumors, including gastric cancer (4.84 ± 0.74 ng/mL), colorectal cancer (4.20 ± 0.68 ng/mL), and oesophageal, pancreatic, or liver cancer (4.67 ± 0.45 ng/mL), with a significant elevation (*p* < 0.001) in comparison with a healthy control group (1.57 ± 0.13). The differences between different types of gastrointestinal tumors were not significant [12].

Chitotriosidase is an enzyme belonging to the chitinase family, involved in the protection against pathogens with a chitin cell wall [13]. Chitotriosidase is considered an important biomarker for inherited lysosomal storage disorders such as Gaucher disease [14]. Its synthesis takes place in both physiological and pathological conditions, predominantly in activated macrophages, neutrophils, Kupffer cells, or bronchial epithelial cells [15]. According to van Eijk et al., chitotriosidase production can be triggered by the granulocyte-macrophage colony-stimulating factor (GM-CSF) [16].

Thein et al. evaluated chitotriosidase and neopterin levels in patients with primary breast cancer and prostatic cancer in different evolutive stages [17]. The diagnostic capacity of the two biomarkers was evaluated with the ROC (receiver operating characteristics) curve. Patients with breast cancer presented a significantly higher chitotriosidase activity in comparison with control females without cancer (*p* < 0.0001). Patients with prostate cancer also presented a significantly higher chitotriosidase activity in comparison with control, cancer-free males (*p* < 0.05). For neopterin values in breast cancer, the differences with gender-matched controls were not statistically significant, though median neopterin was significantly higher (*p* < 0.0001) in prostate cancer subjects in comparison to the healthy males’ group. No significant differences were observed between male and female controls for both biomarkers. For the diagnosis of breast cancer, the AUC (area under the curve) was 0.97, indicating significance (*p* < 0.0001) for chitotriosidase at the cut-off value of 13.80 nmol/mL/h, though not for neopterin (AUC = 0.68, *p* = 0.88). For prostate cancer diagnosis, chitotriosidase presented a significant AUC of 0.64 (*p* < 0.05) at the cut-off value of 13.80 nmol/mL/h, and also neopterin had a significant (*p* < 0.0001) AUC of 0.76, at the cut-off value of 7.6 nmol/L.

Kukur et al. also described a significantly (*p* < 0.05) higher chitotriosidase activity in patients with primary prostate cancer (91.33 ± 8.32 nmol/mL/h), compared to those with biopsy-certified benign prostatic hyperplasia (69.72 ± 8.69 nmol/mL/h). In addition, a higher chitotriosidase activity was observed in the group with a higher Gleason score (118.18 ± 10.28 nmol/mL/h) [18].

This study aimed to evaluate the association of chitotriosidase and neopterin (two novel molecular biomarkers) with tumor pathological characteristics (TNM stadialisation) and prognosis at the presentation of gastric cancer in a surgical department. Our hypothesis was that elevated levels of neopterin and chitotriosidase might be related to more advanced tumors and poor survival rates.

## 2. Materials and Methods

### 2.1. Participants, Setting, and Study Design

The cohort was evaluated in an observational, longitudinal, prospective study. Participants were selected from the patients presenting with surgery indication for gastric cancer at the “Prof. Dr. Octavian Fodor” Regional Institute of Gastroenterology and Hepatology, Cluj-Napoca, Romania, between 8 August 2019 to 28 January 2021, who gave their informed consent for participation. Only thirty-nine patients with gastric adenocarcinoma confirmed by pathology report were included, whereas other types of tumors were excluded.

The sample was divided into multiple subgroups according to the following criteria: resectability (patients with gastrectomy and patients with palliative procedure), survival in the first 30 days and in the first year, the TNM (tumor-node-metastasis) stage, the T (tumor) stage, class, and the presence of metastasis (M1). Neopterin levels and chitotriosidase activity were compared between groups. All patients had at least one open laparotomy and the presence of metastasis was confirmed during surgery and in the pathology report for unresectable cases.

### 2.2. Variables, Data Source, and Collection

Demographic data (age, sex, and urban setting), routine blood test results to evaluate nutritional status, blood group and anemia (albumin, total proteins, and hemoglobin levels), information about the surgical procedure, intraoperative findings, tumor extension or metastasis, as well as pathology reports regarding tumor type and stage, were collected. Data about preoperative neoadjuvant treatment were also considered.

Overall survival represented the interval between the date of surgical intervention and the date of death. To evaluate survival, patients or their contact relatives received 4 phone calls during the follow-up period. For surviving patients, the last information about survival was obtained up to 20 January 2023.

To determine neopterin levels and chitotriosidase activity, fresh blood samples were collected in EDTA vacutainers (4 mL) for each patient at hospital admission, before surgery, after agreeing to participate in the study and signing the informed consent. The samples were centrifuged within 15 min after collection (3000 rpm, 4 °C, 10 min). The separated plasma was stored at −20 °C. Neopterin quantitation was performed using the Neopterin ELISA kit (Wuhan Fine Biotech, China), according to the manufacturer’s instructions. To measure plasma chitotriosidase activity (expressed as nanomoles of hydrolyzed substrate per milliliter per hour—nmol/mL/h) we used an artificial fluorescent substrate (4-methyl-umbelliferyl-chitotrioside), according to the method described by Hollak et al. [19].

### 2.3. Statistical Methods

The statistical analysis was performed with R Commander (R version 4.0.5). To evaluate the distribution of quantitative data, we used skewness, kurtosis, and the Shapiro–Wilk test. Quantitative data were presented as mean and standard deviation or as median and interquartile range. To compare quantitative data for independent groups, we used the Mann–Whitney test. The log-rank test was used to compare median survival time, according to the procedure and the median values of the biomarkers.

The effect of molecular biomarkers on overall survival (OS) was evaluated with Cox proportional hazard regressions; we presented the hazard ratio with the 95% confidence interval, respectively, the *p*-value. To build the univariate models, we used the absolute values of the determined biomarkers and the values dichotomized with medians. Multivariate models were built that adjusted the previous values for the TNM stage and neoadjuvant chemotherapy. Differences were considered statistically significant at a two-tailed *p*-value of less than 0.05.

To find the best cut-off values and the ability of the studied biomarkers in the prediction of advanced tumor stage (T4), the presence of metastasis (M1), and short time survival (at thirty days and at one year after the intervention), we used the ROC (receiver operating characteristics) curves and the maximum Youden index. The AUC (areas under the curve) computed with bootstrap with 95% confidence intervals were also presented.

We hypothesized that neopterin levels (nmol/L) and chitotriosidase activity (nmol/mL/h) values at presentation were increased in advanced cases (i.e., subjects with higher TNM stages and those with unresectable tumors). We also assumed that these values could be related to patients’ short and long-term outcomes (overall survival).

### 2.4. Ethical Statement

Conducted according to the revised Helsinki Declaration of 2000, this research received approval from the “Iuliu Hațieganu” Ethics Committee (no. 121/24.04.2019) and from the Ethics Committee of the “Prof. Dr. Octavian Fodor” Regional Institute of Gastroenterology and Hepatology (no. 8900/10.07.2019). Before the investigation, all the participants agreed and signed the informed consent form.

## 3. Results

Forty-two patients signed the informed consent and were evaluated; three patients were excluded after the pathology result, two of them with GIST (gastrointestinal stromal tumor) tumors and one with a neuroendocrine tumor. The male-to-female ratio was 1.78 (25/14), and 18 subjects originated from an urban area (46.15%). The average age for the sample was 64.3 + 9.97 years. According to blood group, we had 22 (56.41%) A (II) group patients, 8 (20.51%) O (I) group patients, 7 (17.94%) B (III) patients, and 2 subjects (5.12%) with the AB (IV) blood group. Six patients had a negative Rh factor.

Within the chosen sample, 28 patients (71.79%) had benefitted from gastrectomy (11 total and 17 subtotal gastrectomies), and 11 patients (28.2%) had had palliative procedures. One patient (3.57%) had a positive resection margin. Eight patients (20.51%) received neoadjuvant chemotherapy before surgery. Regarding the TNM stage, at presentation, three patients (7.69%) were in stage I, eight patients (20.51%) in stage II, seventeen patients (43.58%) in stage III, and eleven patients (28.2%) in stage IV. At presentation, eleven patients (28.2%) had metastasis. The mortality in the first 30 days after surgery was 7.6% (3/39 patients); two patients, representing 18.18%, had a palliative approach, and one patient, representing 3.5% of the resected group, had a gastrectomy. The survival rate in the first year after surgery was 56.41% (representing 9% of the subjects with palliative surgery and 75% of the cases with resection).

The differences in neopterin levels and chitotriosidase activity, according to the optimal procedure, were not statistically significant. However, the differences regarding nutritional status were important (Table 1). For neopterin values (*p* = 0.07, Mann–Whitney test) or chitotriosidase activity (*p* = 0.82, Mann–Whitney test) no statistically significant differences were observed between patients who benefitted from neoadjuvant chemotherapy and those without preoperative treatment at presentation.

Comparing the values of the biomarkers between the individual T-groups (T1 vs. T2 vs. T3 vs. T4), the differences were not significant for both chitotriosidase (*p* = 0.3977—Kruskal–Wallis test) and neopterin (*p* = 0.15—Kruskal–Wallis test). The differences in the values of the studied biomarkers between the individual N stages (N1 vs. N2 vs. N3) were not significant for either chitotriosidase (*p* = 0.6374—Kruskal–Wallis test) and neopterin (*p* = 0.51—Kruskal–Wallis test). The differences in chitotriosidase activity and neopterin levels, according to the subgroups of different T stages, the presence of metastasis, and the length of survival (30 days and one-year survival), are presented in Table 2.

In univariate analysis, neopterin levels and chitotriosidase activity were not significantly associated with poor survival. For the multivariate model, only the chitotriosidase activity was significantly associated with poor outcomes (Table 3).

**Figure 1:**
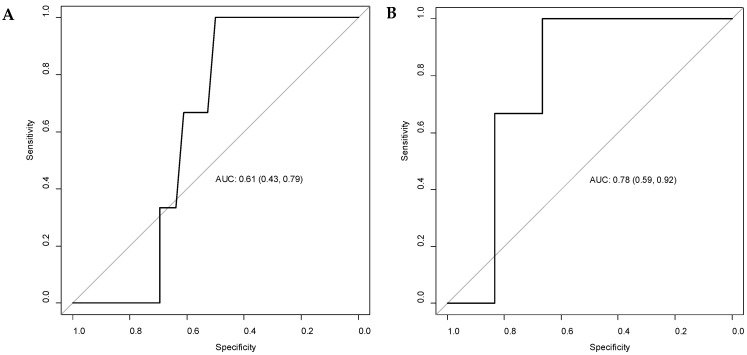
ROC (receiver operating characteristic) curves for neopterin levels (**A**) and chitotriosidase activity (**B**), at presentation, considering patients’ death in the first 30 days after the intervention. AUC = area under the curve, with 95% confidence intervals.

The ROC (receiver operating characteristic) analysis for patients that died in the first 30 days after intervention showed a cut-off value of 10.22 nmol/L for neopterin levels [AUC = 0.61; 95% CI (0.43–0.78)] and 310 nmol/mL/h for chitotriosidase activity [AUC = 0.78; 95% CI (0.61–0.92)] at presentation (Figure 1). For patients who died within the first year after the intervention, the cut-off values were 7.15 nmol/L for neopterin [AUC = 0.54; 95% CI (0.35–0.72)] and 105 nmol/mL/h for chitotriosidase activity [AUC = 0.51; 95% CI (0.32–0.70)]. For the presence of metastasis (M1), the cut-off value for neopterin was 7.15 [AUC = 0.63; 95% CI (0.43–0.81)] and 275 nmol/mL/h for chitotriosidase activity [AUC = 0.62; 95% CI (0.42–0.81)]. For the T4 stage, the cut-off value for neopterin was 16.32 [AUC = 0.66; 95% CI (0.46–0.83)] and 342.5 nmol/mL/h for chitotriosidase activity [AUC = 0.57; 95% CI (0.37–0.77)].

A significant difference in survival was observed between the patients with curative resection (median survival time 309 days), compared to those with palliative procedures (median survival time 159 days, *p* = 0.006–logrank test). No significant difference in survival (*p* = 0.28–logrank test) was observed between patients with neopterin levels below (median survival time 215 days) and above (median survival time 299 days) the sample’s median value (10.06 nmol/L). For the patients with chitotriosidase activity above the sample’s median value (270 nmol/mL/h), the median survival time was 159 days, whereas, for those with chitotriosidase activity below the median value, the median survival time was 282 days, without statistical significance at the logrank test (*p* = 0.93).

## 4. Discussion

This study evaluated two novel inflammatory biomarkers, still insufficiently investigated in gastric cancer, the fifth most common type of cancer worldwide and the fourth cause of death among cancers [1], with a poor prognosis in advanced stages [4,5]. As an Eastern European country, Romania has a high incidence of gastric cancer, according to the Global Cancer Observatory [3]. In our patient sample, the number of male patients was almost double the number of females, in accordance with the higher prevalence of gastric cancer in males described in the literature [1,2]. The predominant blood group in our sample was A (II) for more than half of the subjects (56.41%), while the AB (IV) blood group was less represented (5.12%), corresponding to the findings of Yu et al., who indicated a higher risk of gastric cancer for people with the A blood group and a lower risk for the AB blood group [20].

In our patient sample, the mortality in the first 30 days after gastrectomy was 3.5%, higher than the postoperative mortality described in other studies [4,5]. This can be explained by the high percentage of patients with advanced-stage cancers (43.58% stage III and 28.2% stage IV, due to locally advanced tumors or the presence of metastasis confirmed by pathology reports). Isobe et al. reported first-year and five-year survival rates at 88.6% and 70.9%, respectively, for patients with surgical resection, respectively, and 23% and 5.6%, for unresected cases [5]. Due to the short period of follow up, we were able to evaluate only the first-year survival rate, which proved to be lower, at only 75% for surgical resection patients. For patients with metastasis and palliative surgical intervention, the first-year survival rate was 9%, close to the values found by Katai et al. [4]. As expected, a significant difference (*p* = 0.006—logrank test) was observed between the median survival time for patients with curative resection (309 days) and median survival for those with palliative surgery (159 days).

At presentation, there were no significant differences in the values of the studied biomarkers between the patients that benefitted from curative-intent resection and those with a palliative approach (Table 1). A significant difference was observed in the nutritional status, as patients with advanced gastric cancers that were suited only for a palliative approach presented lower albumin and total protein levels. On the other hand, Hacisevki et al. found a significant (*p* = 0.002) negative correlation between neopterin and albumin levels in patients with gastrointestinal cancer. The authors suggested that neopterin and inflammation could contribute to the alteration of serum albumin and total proteins [12]. For Unal et al. [11], neopterin levels were higher in the group with unresectable gastric cancer, advanced stage, or metastasis, though without reaching statistical significance (*p* > 0.05). On the contrary, in our sample, neopterin median values were apparently increased in patients that qualified for curative resection and not in those with palliative surgery, though without a statistical significance between these variations (Table 1). The potential effect of neoadjuvant treatment over the biomarker values was equally not significant in our study.

Gastric cancer TNM (tumor-node-metastasis) stadialisation for the included subjects was performed according to the 8th edition AJCC (the American Joint Committee on Cancer) staging system [21,22], where the presence of metastasis leads to patient inclusion in stage IV. Tumor invasion in nearby organs (T4b), and the presence of 16 or more positive lymph nodes (N3b), but without metastasis, is indicative of stage IIIC. We have to mention that all palliative cases in our cohort were a direct cause of metastatic disease. Patients with tumor invasion benefitted from curative-intent resection, and only one of them presented R1 positive resection margins, as shown in the results section. The N-stage data were available only for patients with resection and a complete pathology report. This did not influence the TNM stadialisation, as for unresectable subjects, it was attributed according to the presence of metastasis. Considering the low number of subjects, the T classes were also grouped as low (stages T1, T2, and T3) and high (stage T4), with serosa or other organ invasion. We also grouped the TNM stage for COX regressions on the same principle.

Chitotriosidase and neopterin have been previously evaluated together as biomarkers of macrophage activation in infectious diseases such as brucellosis [23], ankylosing spondylitis [24], microvascular complications of type I diabetes [6], and even lung [25], breast [17], and prostate [17] cancer. Chitotriosidase and CHI3L1 (Chitinase-3-like-1 protein) are part of the same family of chitinases [26]. CHI3L1 is more frequently evaluated in the scientific literature. Some tumor-promoting mechanisms were highlighted. CHI3L1 (known also as YKL-40) was associated with angiogenesis and bad prognosis in tumors such as breast, lung, and cervical cancers [27,28,29]. Also, according to the literature, IL-8 (Interleukin-8) and VEGFA (vascular endothelial growth factor) angiogenic properties may be influenced by CHI3L1 to promote cancer progression [30,31,32].

According to Thein et al., significantly higher (*p* < 0.005) neopterin levels were observed in breast cancer patients with metastasis (10.02 nmol/L), in comparison with localized tumors (6.34 nmol/L). The same pattern was observed for prostate cancer, with significantly (*p* < 0.0005) higher median neopterin levels (21.7 nmol/L) for metastatic tumors, in comparison to localized prostate cancer (8.26 nmol/L). The chitotriosidase activity was higher in the group with metastasis, in comparison with localized disease, for both breast and prostate cancer, though without statistical significance [17]. In our cohort, the biomarkers’ values were higher in the groups without metastasis and lower tumor stage (Table 2), but without a significant difference between the groups. This could be attributed to a better immune response in less advanced stages, as well as to the attenuation of inflammatory activation in higher-stage or metastatic cancer. A few studies [11,12,17,18] suggested elevated chitotriosidase activity or neopterin levels in patients with neoplasia and indicated the utility of these molecular biomarkers in the diagnostic process.

On the other hand, Murr et al. argued that neopterin levels at presentation could be relevant for prognosis due to its association with tumor stage, though they also mentioned that this approach would be difficult to use for screening and diagnostic purposes [10]. Due to altered cell surface compared to normal cells, the malignant cells can lead to an activation of the cellular immune system and neopterin release. It is possible that the cellular immune reaction towards the tumor, which may be stronger in individuals with more aggressive tumors, explains why those with higher neopterin production have a higher tumor stage and a poorer prognosis [10]. According to this study, the frequency of elevated serum levels of this biomarker is related to tumor type; it has been observed in 42% of cases with gastric cancer, which is lower than the frequency of increased levels in hematological malignancies, but above that of reported elevations in prostate, breast cancer, or malignant melanoma [10]. The cut-off values were computed in our study for the pathological proof of advanced stages, such as the presence of metastasis and local tumor invasion (T4), however, the values were not statistically significant.

No statistically significant differences in biomarker values were observed between short-term (30 days) and first-year survival (Table 2). In gastric cancer, Unal et al. described a better survival (42.05 months) when serum neopterin levels were below the cut-off value of 11.15 nmol/L, compared to the subjects with serum neopterin values above the cut off (28.53 months, *p* < 0.05) [11]. In our study, the cut-off values for survival at 30 days and at one year after the intervention were computed with the ROC curves and maximum Youden index. Chitotriosidase activity over 310 nmol/mL/h was significant for short-term survival (Figure 1). For one-year survival, the cut-off values of neopterin indicated a poor AUC and no statistical significance. In a multivariate Cox regression, neopterin levels proved to be an independent factor for the prediction of overall survival in gastric cancer, with an HR of 1.052; 95% CI (1.014–1.092), *p* = 0.007 [11]. In the univariate model, the individual effect of the studied biomarkers on overall survival did not reach statistical significance (Table 3).

Due to the low number of subjects, in order to prevent overfitting, the important tumor pathological characteristics were considered together as the TNM stage. Moreover, stages I and II were grouped together, as they were poorly represented. In this model, we appreciated that the maximum number of covariates is two, and we also considered the impact of neoadjuvant therapy. Contrary to the findings of Unal et al. [11], according to our results, neopterin did not prove to have a prognostic value, though chitotriosidase activity presented a significant influence (HR = 1.0038, *p* = 0.03).

This research, however, is subject to several limitations: (one) other comorbidities, treatments, or inflammatory disorders were not taken into account before patient selection; (two) the study was based on a single-center’s experience; (three) the low number of subjects.

Being largely accessible with reduced measuring costs, chitotriosidase, and neopterin may have the potential to improve, not only the diagnostic methods but also the prognostic models in multiple malignant pathologies [11,12,17,18]. According to our knowledge, neopterin and chitotriosidase have not been evaluated yet in Romanian patients with gastric cancer. Our study attempted to evaluate the association of the circulating levels of these molecular biomarkers with tumor characteristics and to establish their prognostic value for patient survival.

## 5. Conclusions

In the present study, neopterin did not display significantly higher values in patients with advanced-stage gastric cancer or poor prognosis; on the contrary, lower values (without statistical significance) were associated with higher T-stage, the presence of metastasis, and poor survival. The chitotriosidase activity was also lower in the group with palliative interventions, without reaching statistical significance, but displayed higher values in the groups with poor survival and was significantly associated with poor outcome, according to the multivariate model regression. We speculate that chitotriosidase may be a useful biomarker to evaluate the prognosis of gastric adenocarcinoma, though larger studies comprising a higher number of subjects are necessary for confirmation.

## Figures and Tables

**Table 1:** Laboratory findings according to the optimal procedure (*n* = 39).

	All Subjects (*n* = 39)	Curative Resection (*n* = 28)	Palliative Surgery (*n* = 11)	*p*-Value
Chitotriosidase (nmol/mL/h)	270.00 (130.00–395.00)	290.00 (137.50–400.00)	230.00 (107.50–297.50)	0.23
Neopterin (nmol/L)	10.06 (5.31–18.15)	10.49 (5.61–20.95)	7.12 (5.31–10.50)	0.21
Total proteins (g/dL)	6.50 (5.70–7.25)	7.05 (6.00–7.50)	5.70 (5.45–6.55)	0.01
Albumin (g/dL)	3.90 (3.60–4.10)	4.00 (3.80–4.23)	3.70 (3.35–3.90)	0.04
Haemoglobin (g/dL)	10.9 (9.3–12.85)	11.35 (10.20–13.38)	10.00 (9.05–11.70)	0.16

Values presented as the median and interquartile range (Q1–Q3); *p* value (Mann–Whitney test) represents the comparison between the group with curative resection and the group with palliative surgery.

**Table 2:** The association of chitotriosidase activity (nmol/mL/h) and neopterin levels (nmol/L) at presentation with tumor characteristics and short-term survival.

Group of Patients (*n* = 39)	Neopterin (nmol/L) Yes*	Neopterin (nmol/L) No**	*p*-Value	Chitotriosidase (nmol/mL/h) Yes*	Chitotriosidase (nmol/mL/h) No**	*p*-Value
T34 vs. T12 (*n* = 23)	8.47 (5.31–18.27)	11.09 (8.43–13.72)	1	280 (125–395)	250 (220–295)	1
T4 vs. T123 (*n* = 29)	7.12 (5.21–11.73)	16.42 (7.25–25.62)	0.1	255 (127.5–333.75)	300 (145–400)	0.45
M1 vs. M0 (*n* = 11)	7.11 (5.31–10.5)	10.49 (5.61–20.95)	0.21	230 (107.5–297.5)	290 (137.5–400)	0.23
Death during the first 30 days (*n* = 3)	7.12 (6.47–8.59)	10.22 (5.21–19.55)	0.54	490 (405–525)	250 (127.5–367.5)	0.12
Death during the first year (*n* = 17)	7.12 (5.33–16.22)	10.22 (5.32–19.67)	0.65	270 (170–360)	270 (130–397.5)	0.91

Yes*—values for subjects with more advanced T or M stage, or death within the first 30 days or the first year, respectively. No**—values for subjects with lower T stage, without metastasis and improved survival. Results are presented as median and interquartile range.

**Table 3:** Univariate and multivariate Cox proportional hazard regressions on chitotriosidase activity (nmol/mL/h) and neopterin levels (nmol/L) at presentation, adjusted for TNM stage and neoadjuvant chemotherapy.

	HR Unadjusted	(95% CI)	*p*	HR Adjusted *	(95% CI)	*p*
Neopterin (nmol/L)	1.002	(0.9659–1.04)	0.9	1.0012	(0.952–1.0523)	0.96
Chitotriosidase (nmol/mL/h)	1.001	(0.9988–1.003)	0.37	1.0038	(1.00023–1.007)	0.03
Neopterin ≥ median	0.62	(0.267–1.48)	0.29	0.52	(0.18–1.46)	0.22
Chitotriosidase ≥ median	1.038	(0.43–2.507)	0.99	3.101	(0.758–12.69)	0.11

HR—hazard ratio; 95% CI—95% confidence interval; * Adjusted for TNM stage (I + II, III, and IV) and neoadjuvant chemotherapy.

## Data Availability

The data presented in this study are available on request from the corresponding author. The data are not publicly available due to restrictions both of privacy and ethics.
